# Electrical and Structural Characterization of Few-Layer Graphene Sheets on Quartz

**DOI:** 10.3390/ma15155330

**Published:** 2022-08-03

**Authors:** Kazybek Aimaganbetov, Nurlan Almas, Bayan Kurbanova, Dauren Muratov, Abay Serikkanov, Zinetula Insepov, Nurlan Tokmoldin

**Affiliations:** 1 Institute of Physics and Technology, Satbayev University, Almaty 050032, Kazakhstan; kazybek012@gmail.com (K.A.); daurenmuratov100@gmail.com (D.M.); serikkanov@gmail.com (A.S.); 2 Department of Science and Innovation, Astana IT University, Nur-Sultan 010000, Kazakhstan; 3 Department of Physics, School of Sciences and Humanities, Nazarbayev University, Nur-Sultan 010000, Kazakhstan; bayan.kurbanova@nu.edu.kz (B.K.); zinsepov@purdue.edu (Z.I.); 4 School of Nuclear Engineering, Purdue University, 400 Central Dr, West-Lafayette, IN 47907, USA; 5 Institute of Physics and Astronomy, University of Potsdam, 14476 Potsdam, Germany

**Keywords:** few-layer graphene, Raman spectroscopy, Hall effect, admittance, scanning electron microscopy

## Abstract

Despite the impressive performance and incredible promise for a variety of applications, the wide-scale commercialization of graphene is still behind its full potential. One of the main challenges is related to preserving graphene’s unique properties upon transfer onto practically desirable substrates. In this work, few-layer graphene sheets deposited via liquid-phase transfer from copper onto a quartz substrate have been studied using a suite of experimental techniques, including scanning electron microscopy (SEM), Raman spectroscopy, admittance spectroscopy, and four-point probe electrical measurements. SEM measurements suggest that the transfer of graphene from copper foil to quartz using the aqueous solution of ammonium persulfate was accompanied by unintentional etching of the entire surface of the quartz substrate and, as a result, the formation of microscopic facet structures covering the etched surface of the substrate. As revealed by Raman spectroscopy and the electrical measurements, the transfer process involving the etching of the copper foil in a 0.1 M solution of (NH_4_)_2_S_2_O_8_ resulted in its p-type doping. This was accompanied by the appearance of an electronic gap of 0.022 eV, as evidenced by the Arrhenius analysis. The observed increase in the conductance of the samples with temperature can be explained by thermally activated carrier transport, dominating the scattering processes.

## 1. Introduction

Due to its high electrical and thermal conductivity, combined with thermal stability, mechanical strength, and chemical inertness, graphene has been considered for use in a variety of applications such as transistors [1,2], power detectors [3], mixers [4], low-noise amplifiers [5], frequency doublers [6], resonators [7], and sensors [8]. In addition, its relatively high transparency in the visible spectral range underlines its potential as a transparent electrode for optoelectronic devices, including light-emitting diodes and solar cells [9]. This is in spite of the fact that, in general, graphene exhibits semimetal properties and a zero band gap [10], which limits its wider use in semiconductor applications. The electrical properties of graphene can be strongly influenced by many factors, including the presence of defects and impurities in its structure, and the type and roughness of the substrate on which it is grown. On the other hand, the unique heat conduction properties of suspended graphene motivated experimental studies on its application as a heat spreader, focusing on the use of different substrates, fabrication methods, and layer numbers. Heat spreaders enable us to significantly increase the effective surface area of heat exchange, therefore dramatically facilitating heat dissipation to cool down electronic systems [11]. However, the in-plane thermal conductivity of graphene decreases significantly when contacted with a substrate. Besides, the poor thermal conductivity is strongly related to the presence of structural defects, layer numbers, crystallinity, and grain size [12].

Graphene usually comes in single-layer (SLG) or few-layer (FLG) modifications. This paper studies FLG transferred onto a quartz slide following etching of the original copper foil substrate. Typical routes for the transfer of graphene to a desired technological substrate include non-electrochemical reaction-based bubble-mediated transfer, the dry transfer route, electro-chemical delamination, the scalable roller-assisted delamination transfer method, and the support-free transfer route [13,14,15,16,17,18]. In this work, the wet transfer approach with an ammonium persulfate aqueous solution acting as an etchant for copper was used. This method has, so far, been considered successful at the laboratory scale. However, it is accompanied by a high chance of contamination, labor intensity, and high cost. The sources of the residue and contamination in this method usually include the sacrificial Cu foil substrate, an etchant used to dissolve the sacrificial substrate and a supporting layer. Among the commonly observed defects of graphene transferred by wet etching are cracks, wrinkles, residues, and contamination [19]. It is generally believed that defects in materials degrade their properties. However, they can also be beneficial when dopants are added to control the type of doping and the carrier concentration [20,21,22,23]. The scattering of electrons in single and few-layer graphene, caused by the impact of the substrate, surface contamination, and the effects of static distortions and phonons is still the subject of extensive discussions [24,25,26,27,28]. In particular, a strong sublinear dependence of the conductivity over a wide range of carrier concentrations at room temperature was observed by the authors of the study [29]. The measurement of the radio frequency conductivity of graphene, implemented at low temperatures using cryogenic equipment, can be used to characterize various scattering mechanisms in more detail. Important insights on defects can be provided by Raman spectroscopy, which may reveal the level and type of its doping, as well as the number of graphene layers [30]. Further, the Hall effect measurements are commonly used to extract the doping carrier density thereby supporting the Raman studies [31,32]. The reliability and validity of this method for measuring both the conductivity and mobility of graphene containing wrinkles and multilayer flakes, that were randomly distributed over the graphene surface, was demonstrated in Ref. [33].

For many applications, it is necessary to have graphene on dielectric substrates [34,35,36,37]. However, the synthesis of graphene with good control of the number of layers and the optimal micropattern directly on dielectric substrates remains challenging [38]. Quartz substrates provide significantly higher light transmission rates and improved thermal stability compared to alternatives such as glass and other polished substrates. In addition, quartz has a high melting point and is chemically inert.

The focus of this study is placed on a combination of electrical and optical measurements using admittance spectroscopy, optothermal Raman spectroscopy, and four-point Hall measurements to investigate the transfer-induced evolution of the FLG properties. This will eventually enable us to assess the performance of the material in real-life applications and help to develop strategies for preserving its remarkable characteristics observed in free-standing films.

## 2. Materials and Methods

*Sample preparation:* Few-layer chemical-vapor deposited (CVD) graphene grown on the backside of an 18-μm-thick Cu foil was purchased from Graphenea Inc., (San Sebastian, Spain). The graphene film was transferred onto a quartz slide according to the following special procedure to avoid damage to the film. The quartz substrate was placed in advance in a Petri dish, which was then filled with a 50 mL aqueous solution of 0.1 M ammonium persulfate (NH_4_)_2_S_2_O_8_ [39]. The graphene-coated copper foil was placed onto the surface of the solution (graphene side up) allowing the copper to be etched. Following the complete dissolution of the copper foil, only a sheet of graphene remained on the surface of the solution. Next, the (NH_4_)_2_S_2_O_8_ solution was subjected to a 10× partial substitution (10 mL) with distilled water via a syringe, whilst keeping the graphene sheet floating on the surface. This step was intended to carefully remove the remains of the copper from the solution. Particular attention here should be placed on avoiding surface waves during the solvent exchange, which can affect the quality of the resulting film. The deposition of graphene onto quartz was performed by carefully removing water via a syringe from the Petri dish. During this step, the substrate (quartz) was placed directly under the graphene layer to obtain a quartz-graphene system. Finally, the film was dried in an oven at 70 °C for 1 h.

*Measurements:* The microscopy images of the FLG layers were obtained using a Crossbeam 4500 scanning electron microscope (SEM). Raman laser-induced heating was applied using a continuous-wavelength (CW) Laser Quantum Torus diode-pumped solid-state 532 nm single longitudinal mode laser beam focused onto the sample down to a spot with a 0.9 μm diameter by means of an OptoSigma 40× numerical aperture (NA = 0.75) (Santa Ana, CA, USA). Simultaneous micro-Raman spectral measurements from laser-heated samples were performed in real-time using a Sol Instruments Nanofinder 30 confocal Raman microscope coupled to an Andor iStar iCCD camera (Oxford Instruments, Abingdon-on-Thames, UK). The spectral resolution for an 1800/mm grating was 1.4 cm^−1^. The pixel size on the iCCD camera and the entrance slit to the spectrometer were 13 μm and 150 μm, respectively. All reported Raman intensities, positions, and linewidths are the results of the Lorentzian fitting.

**Figure 1:**
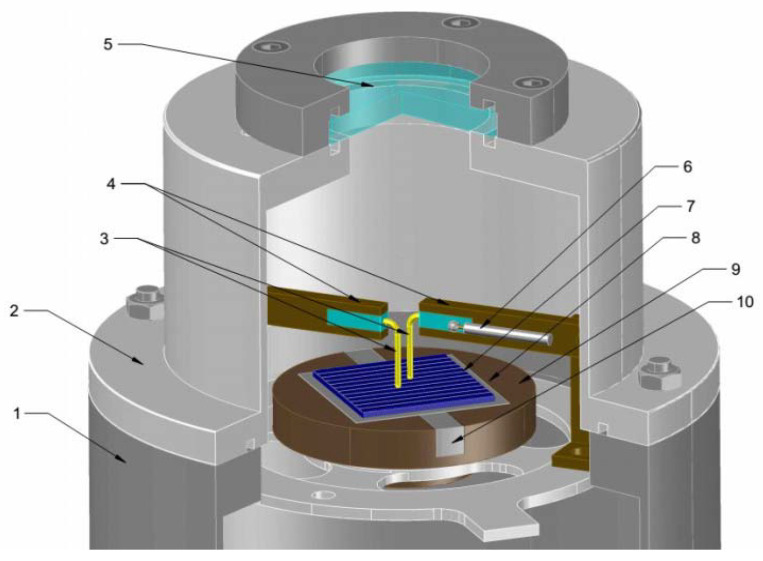
Low-temperature chamber: General view of the admittance measurement chamber: 1—microcryogenic machine body; 2—cell cover; 3—electrical contacts; 4—viewing window; 5—test sample; 6—mica insulator; 7—substrate.

For electrical measurements, the samples were coated with Indium-Gallium contacts at the point of contact with the electrical probes. Four-point probe electrical measurements were performed at room temperature using a Hall effect measurement system HEM-2000 (EGK Corporation, Gyeongsan, Korea). The optical transmission spectra in the wavelength range of 300–1100 nm were taken using a QEX10 PV Measurement setup. Temperature-dependent electrical characterization of the graphene-on-quartz samples was performed using admittance spectroscopy in an evacuated low-temperature chamber between 170 and 350 K [40]. The measurements were carried out in the frequency range of 10 Hz–1 MHz with the amplitude of sinusoidal perturbation of 50 mV. Figure 1 demonstrates the homemade measurement cell and how the sample was mounted onto the cooling base.

## 3. Results

### 3.1. Scanning Electron Microscopy

**Figure 2:**
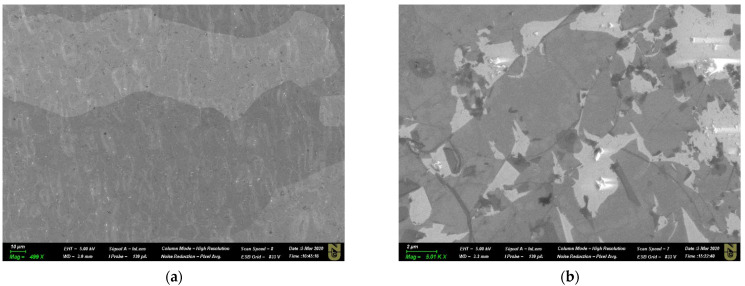
SEM images of (**a**) CVD graphene grown on a Cu foil; (**b**) high-resolution SEM image showing graphene irregularities, such as flakes, wrinkles, and holes.

CVD-grown graphene on Cu [41] often contains a variety of defects, including holes, flakes, wrinkles, Cu grain boundaries, etc. As can be seen in Figure 2, the defects in graphene are located randomly, and their density is quite high. The darker regions in the SEM images correspond to bilayer and multilayer graphene islands formed over a single layer of graphene, which demonstrates good coverage of the surface of the substrate.

**Figure 3:**
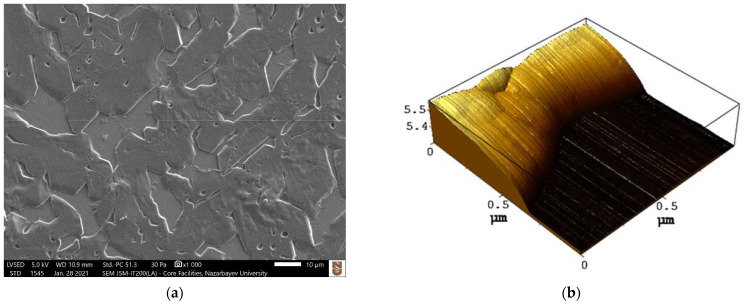

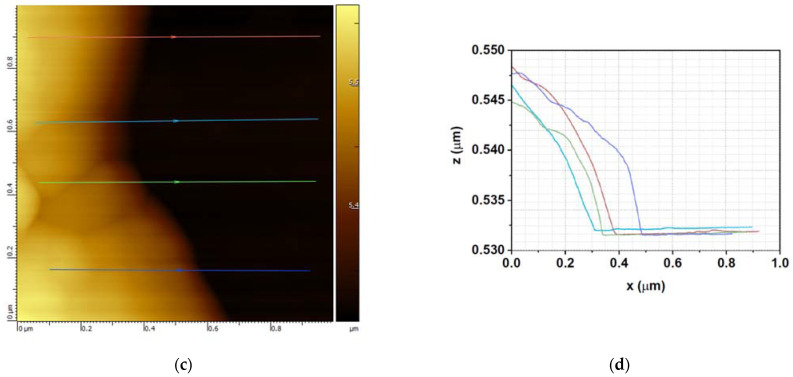
Microscopy images of an etched region of FLG on quartz: (**a**) SEM image; (**b**) 3D AFM image; (**c**) 2D AFM image; (**d**) AFM profiles of the etched quartz regions.

An SEM image of FLG following its transfer onto quartz (Figure 3a) shows microscopic facet structures covering the etched surface of the substrate. The appearance of these structures is related to the anisotropic etching mechanism of quartz. The 2D and 3D images, as well as profiles of the etched region, obtained using atomic force microscopy (AFM), are shown in Figure 3b–d, respectively. Despite the observed irregularities, the film retains continuity and preserves a large proportion of its area.

### 3.2. Raman Spectroscopy

**Figure 4:**
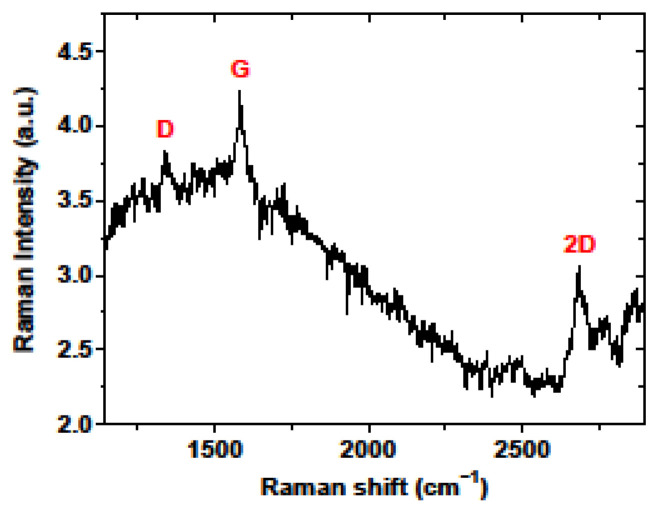
Raman spectrum of few−layer graphene on the copper substrate.

The Raman spectra of FLG on quartz (Figure 4) demonstrate three peaks corresponding to the D (~1350 cm^−1^), G (~1582 cm^−1^) and 2D bands (~2675 cm^−1^). The D-band appears due to lattice disorder [42] and is associated with vibrations of transverse optical phonons at the edge of the Brillouin zone. The G-band is due to first-order Raman scattering by doubly degenerate vibration modes (plane optical transverse and longitudinal phonons) at the center of the Brillouin zone. The 2D-band appears due to second-order Raman scattering on plane transverse optical phonons near the K-point in the Brillouin zone and is closely related to the electronic bund structure [43]. The full width at half maximum (FWHM) of the 2D band has served as an indicator of the number of layers in FLG, according to Ref. [44]. Here we observe the FWHM of the 2D band at 65.7 cm^−1^, which corresponds to four-layer graphene. In addition, the positions of the G and 2D peaks indicate the p-type doping of the graphene. This is consistent with previous findings on the nature and quality of FLG and confirms the validity of the selected transfer approach (see Table 1).

**Figure 5:**
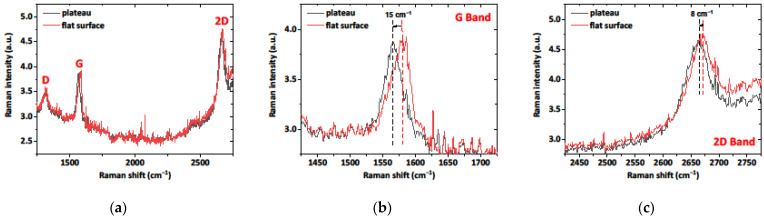
Raman spectra of few−layer graphene on the quartz substrate (**a**), focusing on the G−band (**b**) and 2D−band (**c**) regions, respectively.

As can be seen in Figure 3a, the surface of the sample is significantly distorted due to the non-uniform etching of the quartz substrate. As a result, FLG experiences mechanical strain, which influences the phonon vibrations within the crystal structure of graphene. This can be easily seen in the shifts of the Raman characteristic peaks of graphene [47]. Figure 5 demonstrates the Raman spectra of few-layer graphene on the quartz substrate. Compared to the flat surface, the downshifts in the G− and 2D−bands of graphene are observed for the plateau regions by 15 cm^−1^ and 8 cm^−1^, respectively.

The impact of the strain on the vibrational properties of the crystal lattice is described by the Grüneisen parameter, which can be used to define the biaxial strain through Raman shifts via the following equation [48]:

(1) $$\gamma =\frac{1}{w_{0}}\frac{\delta w}{\delta \varepsilon_{h}},$$

where γ is the Grüneisen parameter for the corresponding G and 2D bands, $w_{0}$ is the Raman frequency without strain (flat surface), δw is the Raman shift, and $\varepsilon_{h}$ is the hydrostatic strain on the graphene films. For biaxial strain, $\varepsilon_{h}=\varepsilon_{t}+\varepsilon_{l}$ and $\varepsilon_{t}=\varepsilon_{l}$, in which $\varepsilon_{t}$ and $\varepsilon_{l}$ are the transversal and longitudinal components of the strain. Based on the Raman shift values of the G and 2D bands and the previously reported corresponding Grüneisen parameter for few-layer graphene [49], the biaxial tensile strain was calculated to be 0.127% from the G band and 1% from the 2D band. The obtained shift-to-strain values are lower than those for the uniaxially [50] and biaxially [51] strained monolayer graphene samples. According to the literature [49], the shift rates of the 2D and G Raman peaks for the bilayer were similar to the monolayer, whereas for the few-layer graphene, a reduction of the above rates was observed possibly due to the cohesive failure within the flakes. As discussed earlier, non-uniform etching leads to the rough surface formation on the quartz substrate. Hence, the substrate exerts the tensile strain on the few-layer graphene through the van der Waals interaction. As the shifts of the Raman bands are directly related to the interatomic force constants, these shifts provide a precise measure of how the stress is distributed within the molecular structure of the few-layer graphene.

### 3.3. Electrical and Optical Measurements

**Figure 6:**
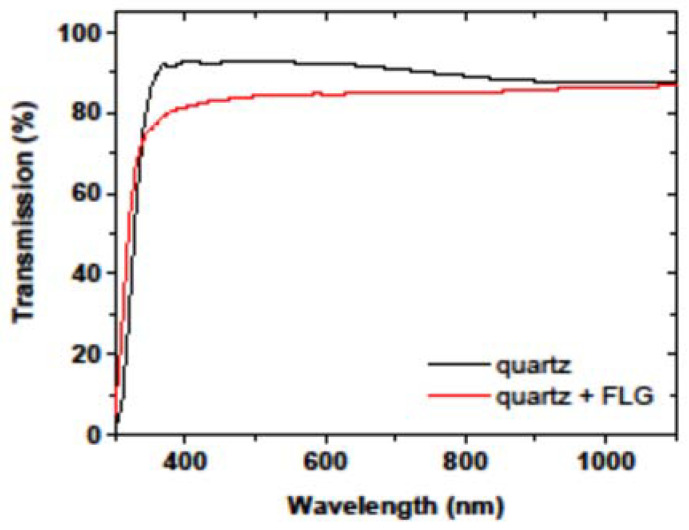
Optical transmission spectra of a quartz slide and the few-layer graphene sample on top of quartz.

Four-point probe Hall effect measurements of the few-layer graphene sample transferred from the original copper substrate onto a quartz slide yielded an apparent p-type sheet doping density of 5 × 10^13^ cm^−2^ and the carrier mobility of 190 cm^2^·V^−1^·s^−1^. Similar p-type doping in few-layer graphene was observed earlier in Ref. [45], which also reported hole mobility of 120 cm^2^·V^−1^·s^−1^. Nevertheless, the sample preserves high optical transparency upon transition onto quartz, as demonstrated by the UV-vis transmission measurements, highlighting the material’s potential for use as a transparent conductive layer (Figure 6).

**Figure 7:**
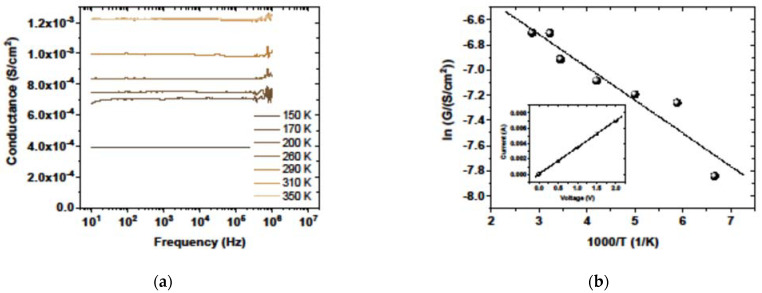
Admittance measurement results: Frequency dependence of sample conductivity at different temperatures (**a**) and the Arrhenius diagram of the logarithm of conductivity versus inverse temperature (**b**) (inset—current-voltage measurements indicating an ohmic contact to the sample).

Further, temperature-dependent electrical characterization of the graphene-on-quartz sample was performed using admittance spectroscopy in an evacuated low-temperature chamber in the temperature range of 170 to 350 K. The linear dependence in the current-voltage measurement of the sample (Figure 7b) indicates the presence of an ohmic transport between the graphene sample and the electric contacts.

The temperature dependence of conductivity is associated with a change in the concentration and mobility of carriers with temperature [52], according to the expression:

*σ* = *σ*_0_ exp(−*E*_a_/*kT*), (2)

where *σ*_0_ is a constant for a given temperature range, *E*_a_ is the activation energy, *k* is the Boltzmann constant, and *T* is temperature. The respective Arrhenius analysis of the temperature dependence of graphene conductivity (Figure 7b) enables us to determine the activation energy of 22 meV. This value is consistent with the results of other authors [53].

The strong dependence of conductivity and charge carrier mobility of FLG and graphene nanoribbons on thickness and temperature was established earlier using theoretical analysis and numerical simulation [54]. The temperature dependence of the conductivity of suspended multilayer graphene was predicted by means of a computational model based on the Boltzmann transport equation and the two-dimensional electron gas theory. According to this work, the increase in conductivity was mainly determined by the reduction in carrier scattering, rather than by the changes in carrier concentration. In our case, on the contrary, the activation of charge carriers from impurity centers with a rising temperature makes a greater contribution to the increase in conductivity, since the degree of doping of the sample is rather high. On the other hand, p-type doping of graphene obtained by etching of the copper substrate has been associated both with the charge impurities present in the etchant and the presence of defects in graphene [55].

## 4. Conclusions

In conclusion, an FLG-on-quartz sample obtained by the etching of copper foil in 0.1 M (NH_4_)_2_S_2_O_8_ demonstrates the appearance of a bandgap, as indicated by the activation energy of 22 meV for conductivity. The microscopic facet structure on the sample surface after transfer from copper foil to a quartz substrate, observed using SEM and AFM, is apparently related to the anisotropic mechanism of substrate etching. The Raman spectral analysis makes it possible to determine the number of graphene layers, as well as the type of doping. Four-point probe Hall effect measurements of the few-layer graphene sample showed an apparent p-type sheet doping density of 5 × 10^13^ cm^−2^ and carrier mobility of 190 cm^2^ V^−1^ s^−1^. It should be noted that the sample preserves high optical transparency upon its transfer to quartz, which was demonstrated by the UV-vis transmission measurements. The sample conductivity shows an increase with temperature, as for a typical semiconductor. This is accompanied by the observation of defects, as evidenced by Raman spectroscopy, which may be introduced into FLG during the transfer process. It is obvious that the presence of impurities may change the density of charge carriers, thereby also influencing the electronic properties of FLG. It is hoped that the results of this study will find use in the development of graphene-based composite materials for electronics.

## Figures and Tables

**Table 1:** Peak positions of the characteristic Raman features of few-layer graphene.

Raman Peak	Peak Position, cm^−1^	Reference
D	1350	[45]
	−	[46]
	1350	Our work
G	1580–1600	[45]
	1593	[46]
	1582	Our work
2D	2660–2700	[45]
	2723	[46]
	2675	Our work

## Data Availability

The data are available by requesting the authors.
